# Development and biomechanical evaluation of a 3D printed analogue of the human lumbar spine

**DOI:** 10.1186/s41205-025-00249-y

**Published:** 2025-01-23

**Authors:** Siril Teja Dukkipati, Mark Driscoll

**Affiliations:** 1 Musculoskeletal Biomechanics Research Lab, Department of Mechanical Engineering, McGill University, 845 Sherbrooke St. W (163), Montréal, QC H3A 0C3 Canada; 2 Orthopaedic Research Lab, Montreal General Hospital, 1650 Cedar Ave (LS1.409), Montréal, QC H3G 1A4 Canada

**Keywords:** Lumbar spine, 3D printing, Benchtop model, Biomechanical testing, Spine mechanics

## Abstract

**Background:**

There exists a need for validated lumbar spine models in spine biomechanics research. Although cadaveric testing is the current gold standard for spinal implant development, it poses significant issues related to reliability and repeatability due to the wide variability in cadaveric physiologies. Moreover, there are increasing ethical concerns with human dissection practices. Analogue models can act as cost saving alternatives to human tissue with better repeatability. The current study proposes a new methodology of spinal biomechanics testing using 3D printable surrogates and characterized its multi-dimensional stiffness in displacement-controlled loading scenarios.

**Methods:**

The model consisted of L1 to S1 vertebrae, intervertebral discs (IVD), intertransverse, interspinous, anterior and posterior longitudinal ligaments. The vertebrae and the IVDs were derived from an open-source 3D MRI anatomography database, while the ligaments were modeled based on literature incorporating mounting points on the spinous and transverse processes. Stereolithography 3D printing along with a combination of stiff and soft photopolymer resins were used to manufacture the vertebrae and the soft tissues in the model. Thereafter, displacement-controlled pure moments were applied in the range of ± 15° at 0.5°/sec in all bending modes using a torsion testing machine and a custom pure bending jig. Model rotation and resisting moment under loading were recorded to quantify the rotational stiffness and hysteresis in the model.

**Results:**

The model reached a maximum of 5.66Nm and 3.53Nm at 15° flexion-extension, 3.84Nm and 3.93Nm at 15° right and left lateral bending, and 2.45Nm and 2.59Nm at 15° right and left axial rotation respectively. Model RMS error against ex vivo human response was estimated to be 1.57°, 1.64°, 0.82° in flexion-extension, lateral bending and axial rotation respectively. Bilateral symmetry in model stiffness was observed in lateral bending and axial rotation directions.

**Conclusions:**

This study presents a reproducible 3D printable L1-S1 lumbar spine and validated it in all three orthogonal bending modes in the range of ± 15° against ex vivo and in silico data. The 3D printed analogue spine model described herein shows promising results, suggesting this model, with further validation, could have potential as a human cadaveric tissue substitute within the explored contexts of use.

## Background

Human and animal cadaveric testing are the current gold standards for spinal implant development. Clinical studies are mandatory for any new spinal system to be approved by most regulatory bodies [1]. Cadaveric testing introduces a high level of inter-specimen variability due to patient medical history and can be problematic to demonstrate any significant difference between performance of two iterations of a spinal implant design, per say. Further, they can be very cost intensive and typically require some level of training to handle the specimens. There is also an argument of ethical considerations involving cadavers for testing [2]. On the other hand, many finite element models of the spine have been reported in the literature, but these studies are limited by the quality and extent of patient anatomical data available [3]. A recent survey on finite element practices of 65 Intervertebral Body Fusion Devices cleared by the US Food and Drug Administration highlighted a lack of standardization and best practices among medical device manufacturers [4]. Moreover, depending on the model complexity, they may require simplifying assumptions such as adopting only linear elastic material models, impacting their context of use. These drawbacks may be overcome by developing reproducible and validated analogues of the human lumbar spine which can be used in conjunction with existing methods of device verification and validation. Specifically, these analogues offer the potential to streamline early-stage design cycles, enabling implant manufacturers to efficiently test and compare design iterations in a cost-effective, controlled and repeatable manner.

The development and use of analogue lumbar models is gaining popularity amongst researchers. A full lumbar spine surrogate has been developed and validated by Sawbones Inc. (WA, USA) and is currently the only commercially available model on the market for researchers [5–11]. This is perhaps due to challenges in mimicking the complex anatomy while keeping the material and design choices relevant for current manufacturing techniques. This model was composed of a polyester fabric mesh embedded in polyurethane resin matrix with varying lay-up orientations. These material choices and fiber orientations were determined through extensive iterative testing to match model material properties with human tissue data [6]. After extensive testing and fine-tuning, this model has been validated in flexion-extension (F-E), lateral bending (LB) and axial rotation (AR) and can generate moments within one standard deviation of a healthy human spine response in pure bending mode [8]. Another approach to develop such a model could be incorporating the use of 3D printing for manufacturing the models, which is time and cost-efficient.

Historically, 3D printing has been used to develop models of patient-specific spinal anatomy through MRI scan data for surgical planning and educational purposes [12–14]. Recently, Bohl et al. led an effort to develop a single stage lumbar analogue model using 3D printing [15–17]. Called the Barrow Biomimetic Spine, this model used Fused Deposition Modelling type 3D printing and was able to demonstrate that carefully tuned print parameters can in-fact produce analogue models with reasonable mechanical stiffness and range of motion comparable to their human counterparts [16]. Likewise, Franceskides et al. used 3D printing to generate patient-specific single stage analogue models to test the feasibility of employing 3D printing and soft materials to fine-tune the analogue model stiffness response [18, 19]. The validation of such an analogue model was done by comparing the model stiffness response under loading to historically available human ex vivo data.

Many such ex vivo biomechanical tests on human lumbar spine segments have been reported in literature. For this study, a comprehensive review of these studies was conducted and those employing pure bending loading on the lumbar spine were selected as comparators. Notably, Stubbs in 2011 carried out ex vivo experiments in F-E motion involving various loading scenarios, including pure bending and measured the rotational stiffness of lumbar spines [20]. Likewise, Rao in 2012 conducted a series of ex vivo experiments in all three pure bending modes to measure the rotational stiffness of fresh, intact human lumbosacral spines [21]. Rao also developed a finite element model with tuned material properties, which was subsequently validated against the experimental data he previously collected. Building on Rao’s work, Campbell et al. in 2016 developed an automated patient-specific finite element pipeline based on MRI scans and validated it against Rao’s experimental data [22]. These aforementioned studies focused solely on the L1-S1 section of the spine and provided ex vivo and in silico validation data for this work. Previous studies have also shown that spinal stiffness varies with loading rate [23] as well as the control strategy used to apply spinal loads, specifically load-controlled and displacement(rotation)-control strategy [24].

Despite these advances in spinal biomechanical characterization and a clear need for analogue models, there are currently no validated L1-S1 analogue models reported in literature, aside from our previous efforts to develop such a 3D printable model [25]. Although Sawbones Inc. has developed L2-L5 and T12-S1 surrogates and validated them against healthy human ex vivo data, their manufacturing process remains highly manual leading to increased cost [7, 26]. Incorporating 3D printing could reduce human error, lower manufacturing time and decrease the cost per model. As a precursor to this study, we also developed and validated single and multi-stage 3D printable analogue models under load-controlled loading scenarios [25, 27]. The purpose of the present study is to build upon this work by developing a fully 3D printed L1-S1 analogue spine model and evaluate its displacement-controlled loading response characteristics in F-E, LB, and AR bending modes.

**Fig. 1:**
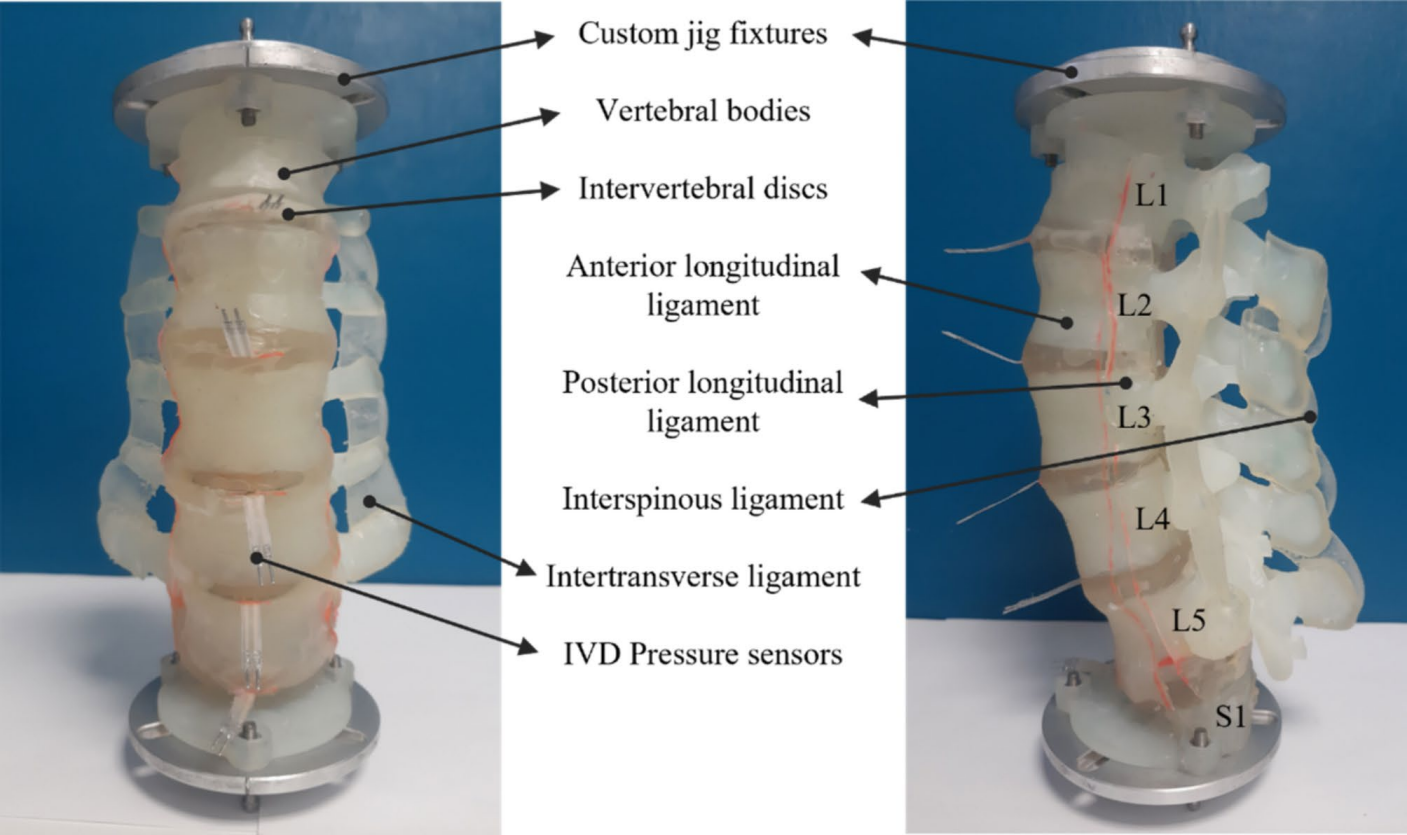
Developed 3D printed analogue spine model

## Methods

### Model creation

The 3D models of the vertebrae and the IVDs were acquired from 2 mm interval 3D MRI images of a healthy 22-year-old male obtained from “BodyParts3D/Anatomography” - an anatomography database developed by the Database Center for Life Sciences of Japan’s Ministry of Education [28]. The subject was 172.8 cm tall, weighed 65.0 kg, and had a body mass index close to the Japanese average for males aged 18–30 (171.4 cm and 63.3 kg) [29]. The model lordotic angle was measured to be 44.76°, aligning closely with the reported average lordotic angle of 47 ± 10.07° observed in asymptotic younger population [30]. The ligaments were modeled based on lumbar anatomy to accommodate their mounting points on the vertebral bodies. For the interspinous ligament, the spinous process ends of adjacent vertebrae were connected using an interspinous ligament cross section area of 2 mm x 20 mm. Similarly, the intertransverse ligaments were modeled using a cross section area of 2 mm x 12 mm cross-section, connecting the endpoints of the transverse processes. For the anterior and posterior longitudinal ligaments, a cross-section of 2 mm x 20 mm was used along the length of the spine. These cross-sectional dimensions were chosen to match the stiffness of the 3D-printed ligaments with ex vivo specimen data from the literature [31]. The model consisted of L1-S1 vertebrae with L1 and S1 fixed to mounting plates, their corresponding IVDs, intertransverse, interspinous, anterior and posterior longitudinal ligaments as depicted in Fig. 1. These models were then sliced, and 3D printed on Form 3 L (Formlabs Inc, MA, USA) which uses the stereolithography printing technique. The vertebrae were printed with Durable V2 resin, and all the other soft tissues were printed with Elastic 50 A V1 photopolymer resin (Formlabs Inc, MA, USA). Post printing, all the individual models were cleaned in 90% Isopropyl alcohol for 30 min to dissolve any excess resin. Following that, the vertebrae were cured for 60 min while the soft tissues were cured for 15 min under 405 nm UV light on a turntable for even exposure as recommended by the manufacturer. These parts were then bonded together manually with a mixture of 50% w/w Elastic 50 A V1 + Durable V2 mixture. Excess resin was manually removed. The whole model was then cured again in UV light for 45 min completing the manufacturing process.

### Test setup and protocol

**Fig. 2:**
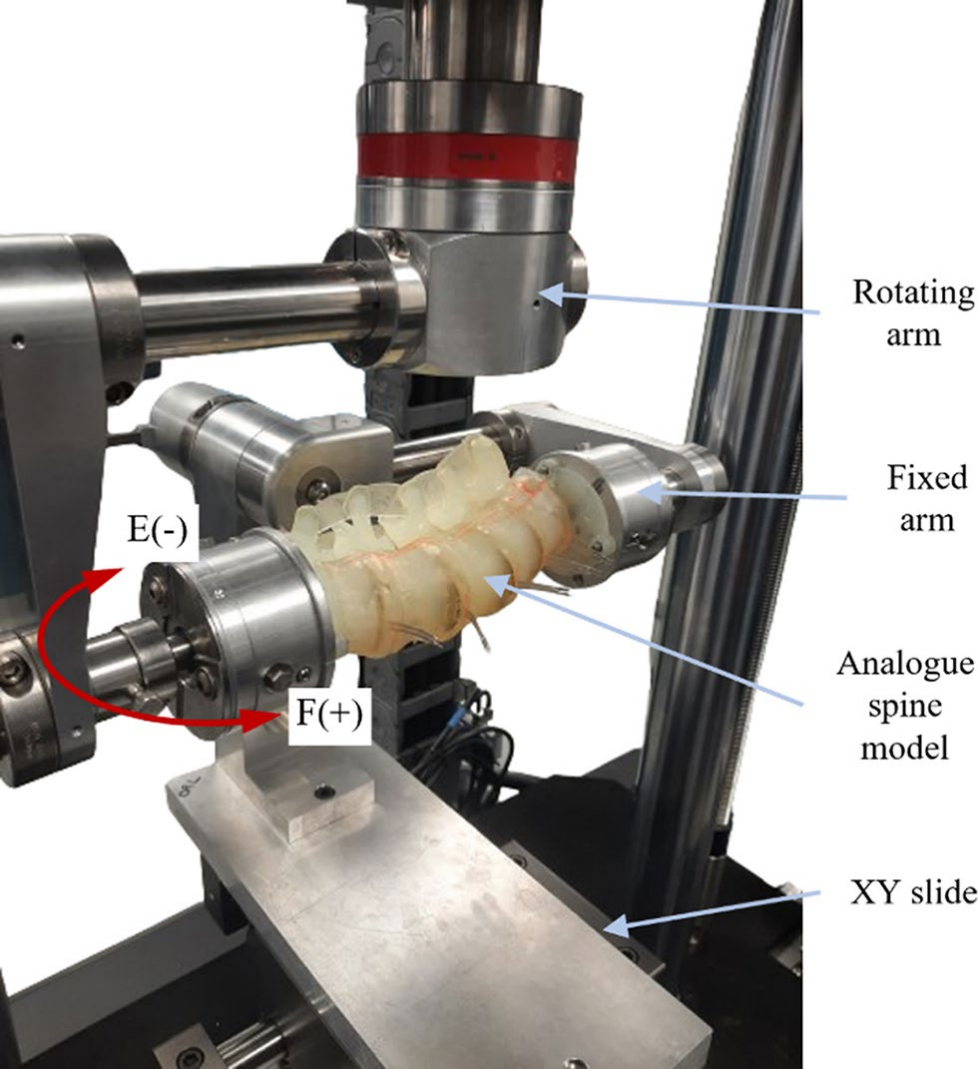
Experimental setup of the developed L1-S1 analogue model in flexion (F) and extension (E). Positive and negative signs indicate sign convention for moment directions

**Fig. 3:**
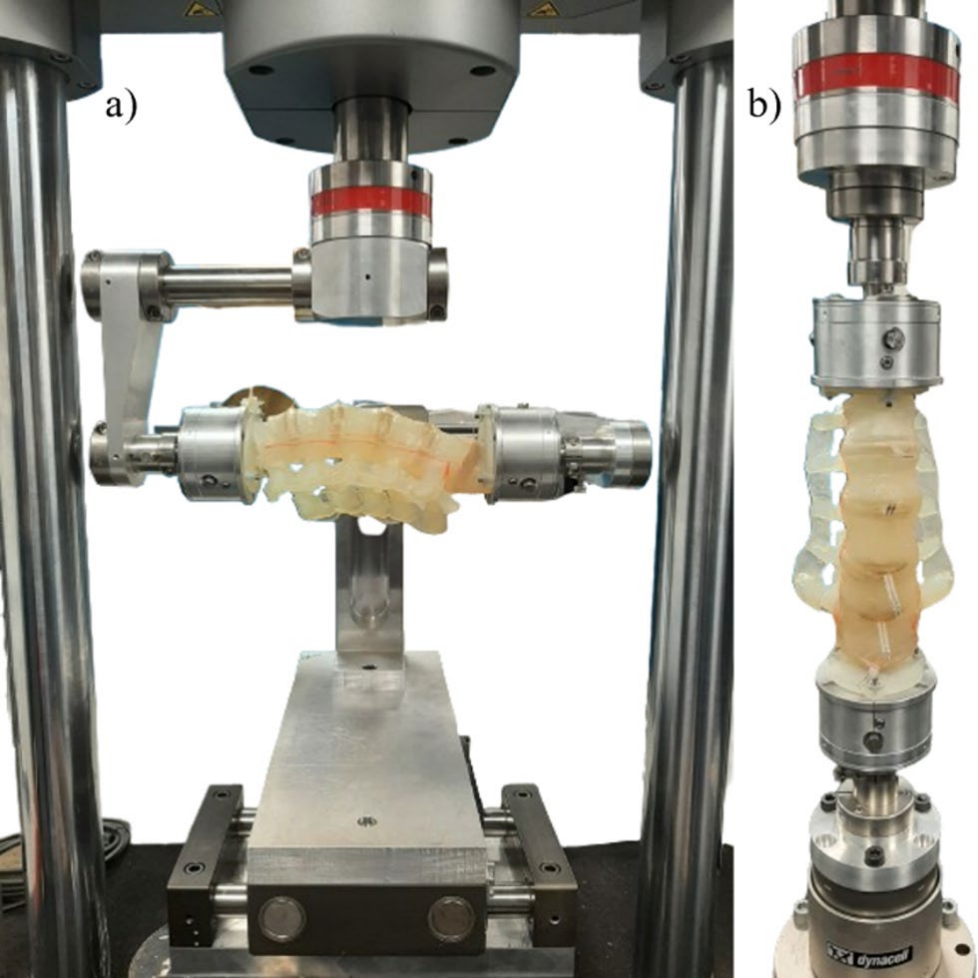
Experimental setup of the developed L1-S1 analogue model in (**a**) LB and (**b**) AR

The analogue model was mounted on a uniaxial testing machine (ElectroPuls E10000 Linear- Torsion, Instron, MA, USA) using a custom bending jig to impart pure bending moment on the model as shown in Figs. 2 and 3. This jig ensured that no off-axis loading was imparted on the model and that the model finds its instantaneous axis of rotation on its own under loading using an unconstrained XY sliding table in F-E and LB [32]. The S1 vertebra was fixed to a load cell (Dynacell™, Instron, MA, USA) through the fixed arm of the jig. The L1 vertebra was imparted a rotational displacement through the rotating arm to measure the resisting bending moment produced by the model. In AR, the model was directly mounted on the testing machine without the bending jig as depicted in Fig. 3b. The setup offered a full-scale range of ± 100Nm and ± 135° deflection with a resolution of ± 0.05Nm in the applied load range. The custom bending jig was assumed to be rigid in the range of applied loads. The model was subjected to 5 cycles of pure bending of ± 15° at 0.5°/sec per test set. A total of four test sets were conducted at least one hour apart, allowing the material to recover from loading. The upper and lower load limits are set to be ± 7.5Nm as a safety precaution. Loading was stopped if the moment exceeded 7.5Nm on either side or if there was any apparent visual damage in the model. No follower load was applied on the model during the experiment.

Data was recorded at 100 Hz across all the test sets. The model resisting moment was plotted against rotational displacement for model bending stiffness. All four test set responses were averaged to find the average model stiffness. Noise in the data was filtered through a curve fitting approach. A four-degree polynomial was fitted through the hysteresis curve and the average stiffness as a function of rotation was determined.

## Results

The model exhibited a hysteresis response under loading and unloading. Moreover, the stiffness response of the 3D printed model is nonlinear and sigmoidal in shape over the range of motion tested. This is also a characteristic of human lumbar segments in bending and is well documented in literature [33]. The model also demonstrated excellent repeatability and has a standard deviation of 0.02Nm in flexion, 0.05Nm in extension, 0.17Nm in LB, and 0.07Nm and 0.03Nm in right and left ARs respectively over 20 cycles. No statistical analysis was performed as the data from only one L1-S1 3D printed model was available even with repeated measures as described. Stiffness curves for the full L1-S1 segment were produced and were not evaluated for individual spinal levels. Root Mean Square Error (RMSE) metric was used to analyze the accuracy of the model by comparing the stiffness measurements with human ex vivo [20, 21] and in silico [21, 22] responses from literature.

### Flexion-extension

**Fig. 4:**
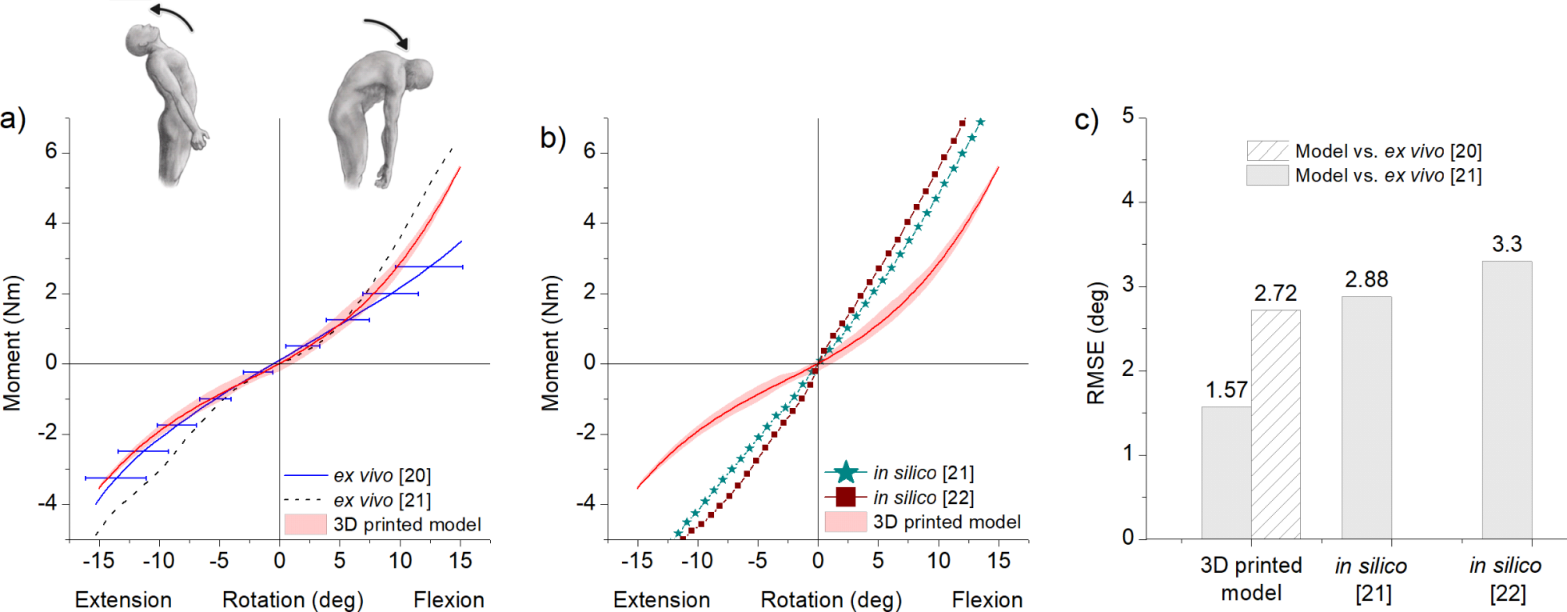
Moment-rotation curves of the 3D printed model in F-E motion compared to similar (**a**) ex vivo [20, 21] and (**b**) in silico [21, 22] studies. (**c**) RMS error between the indicated models and the target response of Rao’s 2012 ex vivo study [21]. Shaded region in the 3D printed model response indicates the loading-unloading hysteresis curve of the model, and the bold line in red indicates the average response. Error bars indicate one standard deviation

The model reached a maximum of 5.66 ± 0.02 Nm at 15° flexion and 3.54 ± 0.05 Nm at 15° extension. For validation purposes, the model’s stiffness response was compared to L1-S1 ex vivo and in silico stiffness responses from literature. When compared to Rao’s 2012 ex vivo study [21], the 3D printed model has an RMSE of 1.57° and 2.72° when compared to Stubbs’s 2011 ex vivo experiments [20]. Both these values were lower than the reported 3.30° RMSE for Campbell et al. finite element model [22] and computed 2.88° RMSE for Rao’s 2012 finite element model [21]. No scaling for model response was applied in F-E. The 3D printed model response was inside one standard deviation band of the human response as shown in Fig. 4. It should be noted that the moment-rotation curve of Stubbs’s 2011 ex vivo response was reconstructed from averaging individual specimen (*n* = 6) moment-rotation curves reported in that work in pure bending mode [20].

### Lateral bending

**Fig. 5:**
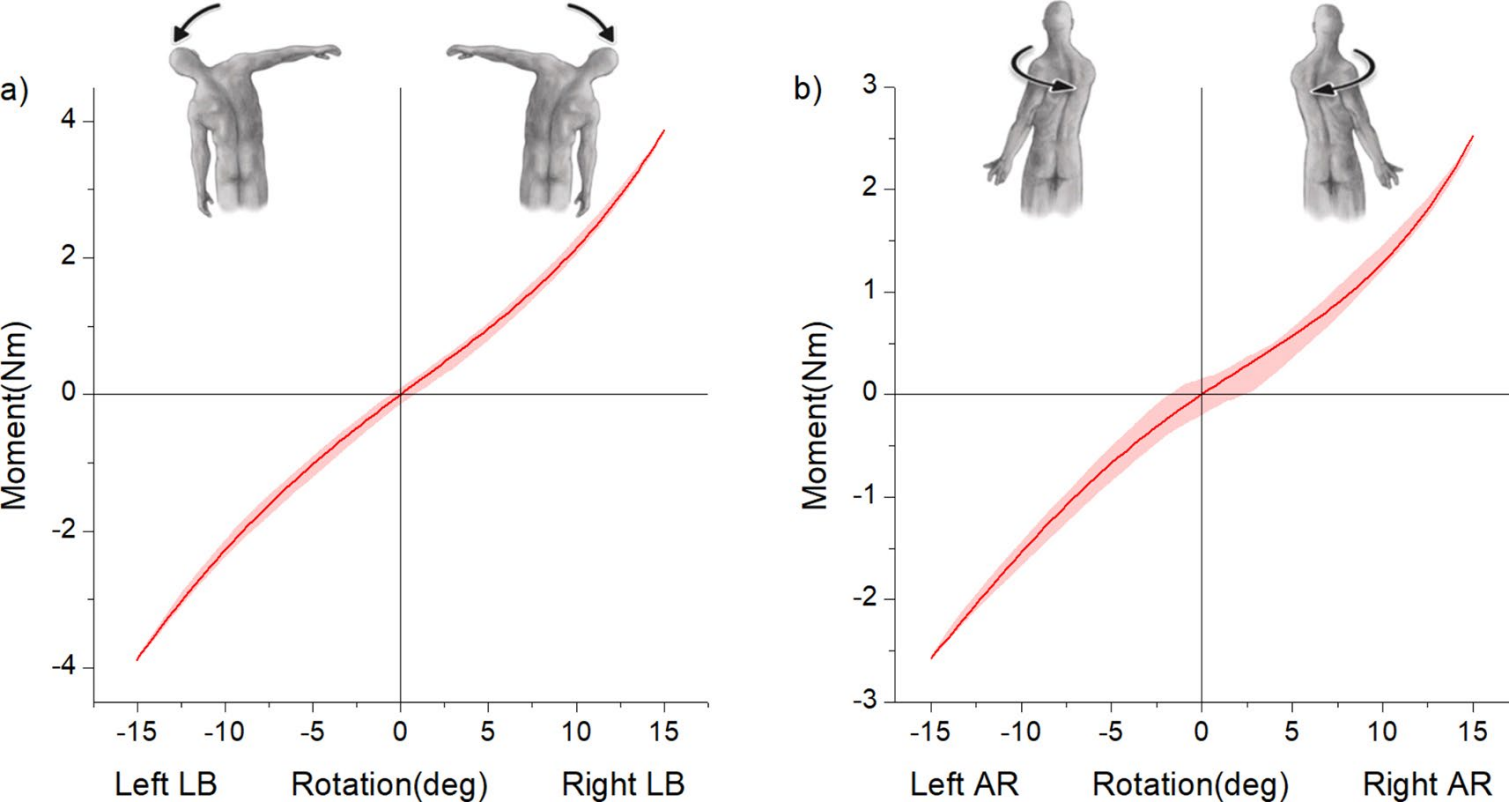
Moment-rotation curves of the 3D printed model in (**a**) LB and (**b**) AR motions

In LB, the 3D printed model was found to be approximately 1.67 times less stiff than its human counterpart with a maximum average moment of 6.6Nm at 15° rotation [22]. A maximum moment of 3.88 ± 0.17 Nm at 15° right bending and 3.95 ± 0.17 Nm at 15° left bending was recorded in the model. Nevertheless, the nonlinear shape of the moment rotation curve was very comparable to literature ex vivo [21] and in silico [22]studies. The model exhibited high degree of bilateral symmetry as depicted in Fig. 5a.

### Axial rotation

In AR, the model recorded a maximum moment of 2.46 ± 0.07 Nm at 15° right rotation and 2.60 ± 0.03 Nm at 15° left rotation, which is approximately three times less stiff than the ex vivo response of 7.5Nm at 15° rotation [22]. Similar to the LB response, the shape of the moment-rotation curve in AR followed a nonlinear hysteretic path much like the human ex vivo spine response. Bilateral symmetry was evident from model response in Fig. 5b.

### Effect of loading rate

**Fig. 6:**
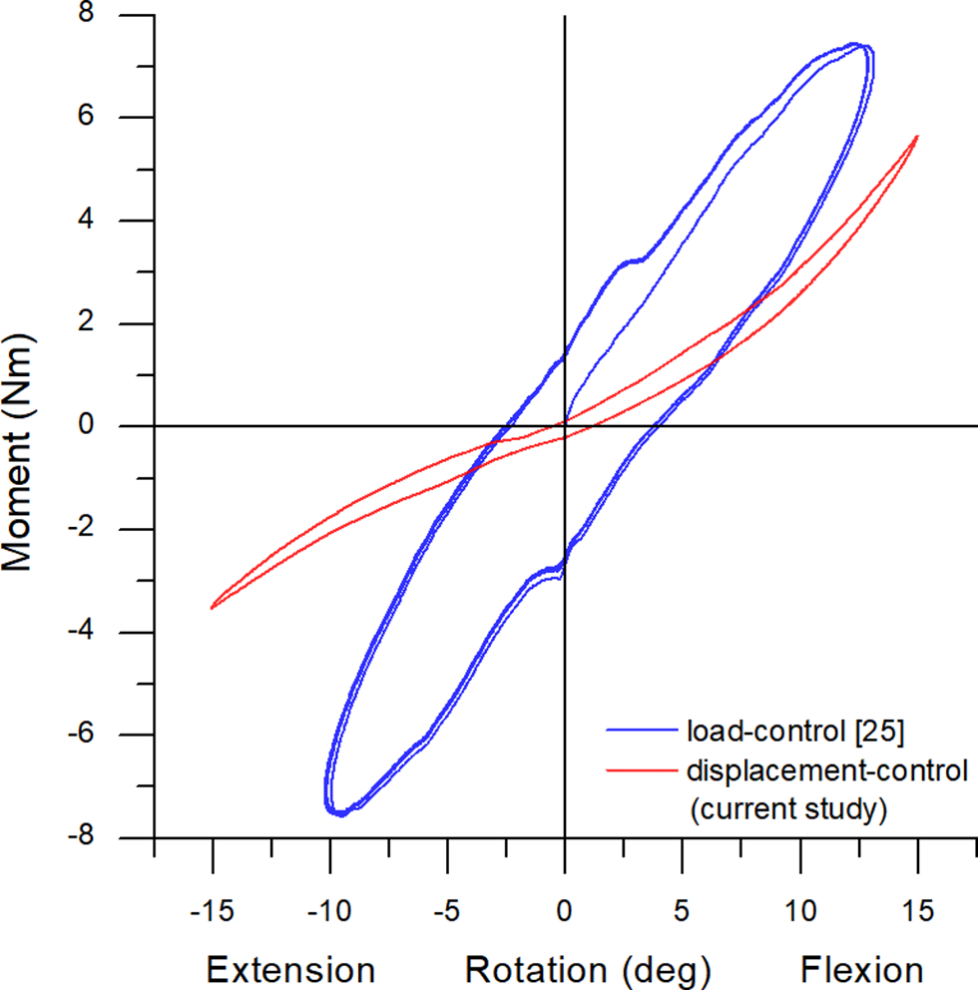
Comparison between load-controlled and displacement-displacement controlled pure bending loading scenarios in the current 3D printed model and one from [25]

A clear change in the degree of hysteresis was observed between the load-control method in [25] and the displacement-control method used to apply pure moment loads in the current study as depicted in Fig. 6. In displacement-control method, a relatively low area between the loading and unloading curves was observed in all three bending directions as opposed to a considerable separation between the loading and unloading curved in the load-control method. The curves were also non-smooth with perturbations in between in the load-control method as opposed to smooth curves in the displacement-control method. These differences could be attributed to the fact that the strain rate in the model was constant in displacement control whereas it is nonlinear in the load-control method. The area under the graph is a measure of mechanical hysteresis in the model and is analogous to the amount of damage per loading cycle in the model due to applied loads.

## Discussion

This study presents the displacement-controlled stiffness characteristics of the developed reproducible 3D printed L1-S1 spine analogue model in pure bending mode in F-E, LB, and AR. Furthermore, the model response is compared to previously published data towards validation and support of its credibility as a possible surrogate to other conventional test platforms. At low loads, the 3D printed model produced a stiffness range that is in good agreement with both experimental and simulation data in terms of its intact stiffness [20–22]. Although the 3D printed model response in F-E motion was comparable to ex vivo and in silico responses, further validation studies for repeatability and sensitivity analysis should be conducted prior to using it as an adequate representation of the human lumbar motion segments. In LB and AR, the model with its current material selection is comparatively less stiff than its human counterpart prompting the use of stiffer photopolymers. Moreover, the 3D-printed model was based on spinal geometry from a 22-year-old healthy male [28], whereas the ex vivo comparison models used for validation were derived from a 33-year-old healthy male [21]. Since a direct comparison of spinal geometries between the studies was not possible, this difference may have contributed to the discrepancies observed in the results.

Sawbones Inc. developed lumbar analogue models of T12-S1, L2-L5 and single stage models that are both anatomically and mechanically correct [7, 26]. These models are currently being used by many researchers to study spinal biomechanics and are the only analogue validated models of the lumbar spine available in the market. Additionally, their construction involved complex fiber weaves and patterns embedded in different types of resins increasing their cost of manufacturing. This study proposes a similar model that aims to achieve a stiffness response comparable to that of the human segments. The construction of the model involved commercially available 3D printable materials and computer-aided design models from an open-source database for easy reproducibility. While this model is cost-effective compared to cadaveric models, it relies on a relatively expensive form of 3D printing using photopolymer resins to manufacture the model. It requires access to a resin 3D printer, the materials and post-processing equipment, which can involve significant capital costs. For researchers without such equipment, outsourcing the printing to a local print shop may be a feasible alternative. Nonetheless, the introduction of 3D printable surrogates could facilitate rapid visualization of spinal biomechanics and may also be used for destructive testing. As Sawbones Inc., models only have T12-S1 and L2-L5 model responses, they were not included in comparisons in this study.

Many researchers have conducted spinal in vivo, in vitro or ex vivo, and in silico testing in the past. The concept of a follower load along the curvature of spine remains a plausible manner to best load excised spines missing all stabilizing tissues, active and passive, in a living person. How this is best incorporated into laboratory tissue testing methods is still unclear due to the lack of understanding of true loading on the lumbar spine. Panjabi et al. [34] adopted a value of 100 N in flexion extension motion while Okawa et al. suggested a value of 182 N [35]. Patwardhan et al. evaluated the dependency of a compressive follower load on the moment-rotation response of the human lumbar spine and found out that the application of optimized follower load can in-fact simulate physiologic compressive loads [36]. Wilke et al. published recommendations for standardizing spinal ex vivo testing in 1998 which are accepted as best practices by many researchers [37]. The current study follows those recommendations although it does not employ any follower load in any bending mode. But with some design changes, it is possible to incorporate cable driven follower loading in the model to simulate physiological in vivo loads in the future if necessary [38]. For future studies, it would be of interest to investigate the effect of a follower load on the rotational stiffness of this model.

To truly understand the stiffness response of a spine motion segment, a load-controlled loading strategy is appropriate as it is representative of the physiological loading on the human spine in vivo. However, a displacement-control strategy was applied in this case as opposed to load-controlled to evaluate model hysteretic behavior and end ranges of motion. Displacement-control also offered a mode of safety during testing as the strain rate of loading could be accurately controlled. This was particularly helpful as previous testing demonstrated the current model’s inability to withstand moments greater than 6Nm. In a load-controlled scenario, there is minimal control over model strain rate which is an important factor in viscoelastic failure of elastomers. The model reached a maximum of 5.66Nm and 3.53Nm in flexion and extension, 3.84Nm and 3.93Nm in right and left LB, and 2.45Nm and 2.59Nm in right and left AR, respectively. These loads are lower than the advised 7.5Nm of physiological load by Wilke et al., [37] and could be attributed to the use of softer Elastic 50 A V1 resin for the IVDs. In future studies, incorporating a stiffer Flexible 80 A resin could improve the load bearing characteristics of the model.

The low RMSE values show that the 3D printed model was able to produce results comparable to ex vivo and in silico human responses in F-E motion. Very low standard deviations were observed in the test cycles indicating superior model repeatability. This model was developed keeping in mind its context of use, i.e., in the range of ± 15° in all flexion-extension motion and as such is validated in this range only. Care must be taken to account for these ranges and loading direction when working with this model.

It is the author’s belief that incorporating anterior and posterior longitudinal ligaments had a significant impact on the model’s F-E stiffness. This observation also supports the fact that the model was less stiff in LB and AR and needed scaling factors due to the absence of soft tissues orienting specifically in those directions. The thickness of the intertransverse ligaments could be increased to increase the LB stiffness which could be easily integrated onto the model. For future iterations, it would be interesting to study the incorporation of fibers and the effect of their directionality on model rotational stiffness. Additionally, a limitation of this study is the absence of facet joints, which are known to contribute to lumbar spine biomechanics [39]. The vertebrae and the IVDs were solid, with no internal anatomical features included in this iteration of the model. Future studies could explore the possibility of using resins with different elastic moduli to 3D print nucleus pulposus and annulus fibrosus within the IVDs, tailoring the disc properties to simulate degenerative cases. Similarly, differentiating the trabecular and cortical bone regions within the vertebrae could enhance the model’s anatomical accuracy and biomechanical mechanical response. In the ever-evolving field of spinal biomechanics, it is important to minimize the use of cadavers in clinical studies. An effort is made to develop an alternative testing platform without ethical, cost and time burdens. The proposed novel model shows promising initial results towards the development of a full-scale lumbar analogue model with stiffer 3D printable materials. It is to be noted that this platform in its current state cannot be used for any safety or approval tests of implants.

Only the response of the full lumbar spine was presented in this study which is not sufficient to fully characterize the spinal biomechanics [40]. Future studies plan to include a level-by-level rotation of motion, and global and local instantaneous axis of rotation tracking to evaluate the contribution of different spinal levels in global segmental rotation. Due to the inherent nature of 3D-printed models, measuring stress and strain fields under loading, as done in finite element models, is not feasible. However, using flexible strain gauge sensors on the soft tissue 3D prints could allow for surface strain measurements. Moreover, incorporating a parametric spine model using statistical shape modeling in addition to the current workflow of using MRI images to derive spinal geometries could also greatly increase patient-specific adaptation of the model. Model response under only pure bending loading has been evaluated in this study. Future studies could focus on validating the model in compression and combined loading scenarios to increase model robustness. Although the manufacturing process and model preparation methods are independent of external factors like the skill of manufacturer, order of assembly etc., future studies will seek to focus on sensitivity of these factors as well as statistical significance of the model stiffness response with higher sample sizes. Multi-resin polyjet printing could enable the inclusion of smaller anatomical features, such as facet joints, and completely eliminate the need for manual assembly in the model manufacturing process.

## Conclusion

This study provides a proof-of-concept of feasibility of developing 3D printable analogue spine models by evaluating the displacement-controlled stiffness characteristics of the developed L1- S1 spine model in F-E, LB, and AR pure bending modes. Furthermore, the model response was compared to previously published data towards validation and support of its credibility as a possible surrogate to other conventional test platforms. At low loads, the 3D printed model produced a stiffness range that is in good agreement with both experimental and simulation data in terms of its intact stiffness in F-E motion. A high degree of repeatability was also observed in the model owing to its low standard deviation across the loading range. Bilateral symmetry was evident in LB and AR motions. Future studies should focus on evaluating statistical significance of model stiffness response with higher sample sizes and sensitivity to external factors like skill level of the manufacturer. Furthermore, incorporation of 3D printable lattice structures to simulate conditions like osteoporosis or vertebral tumors in the vertebral bodies could be a value addition to the model. This open-source model serves as a proof of concept for cost and time-effective high-fidelity biomechanically accurate fully 3D printable analogue lumbar spine models to be used in conjunction with existing cadaveric and finite element methodologies of implant testing.

## Data Availability

No datasets were generated or analysed during the current study.

## Abbreviations

F-E: Flexion-extension
LB: Lateral bending
AR: Axial rotation
IVD: Intervertebral disc
RMSE: Root mean square error
